# Lumbo-sacral epidural anaesthesia as a complement to dissociative anaesthesia during scrotal herniorrhaphy of livestock pigs in the field

**DOI:** 10.1186/s13028-015-0124-0

**Published:** 2015-06-24

**Authors:** Carl Ekstrand, Marie Sterning, Love Bohman, Anna Edner

**Affiliations:** Department of Clinical Sciences, Faculty of Veterinary Medicine and Animal Science, Swedish University of Agricultural Sciences, PO box 7054, SE 750 07 Uppsala, Sweden; Department of Biomedicine and Veterinary Public Health, Faculty of Veterinary Medicine and Animal Science, Swedish University of Agricultural Sciences, PO box 7028, SE 750 07 Uppsala, Sweden; Department of Sociology, Stockholm University, SE 106 91 Stockholm, Sweden

**Keywords:** Ketamine, Detomidine, Lumbo-sacral, Lidocaine, Surgery

## Abstract

**Background:**

In Sweden, scrotal or inguinal herniorrhaphy of livestock pigs in the field has traditionally been an important part of the surgical skills training of veterinary students. Few substances meet the legal requirements for field anaesthesia of production animals in the European Union but a protocol based on azaperone-detomidine-butorphanol-ketamine does. Unfortunately the anaesthesia is characterised by unpredictable duration and depth and of abrupt awakenings which is not acceptable from an animal welfare perspective and impedes surgical training. Lumbo-sacral epidural analgesia is proven to provide sufficient analgesia to allow abdominal surgery, but there are few reports on the field use of this loco-regional technique. The study aim was to evaluate whether lumbo-sacral anaesthesia can be safely and successfully used in the field by a veterinary student and whether the combination of dissociative and lumbo-sacral epidural anaesthesia improves analgesia and anaesthesia to guarantee animal welfare during herniorrhaphy in livestock pigs, enabling surgical skills training.

**Results:**

Pigs in the control-group (placebo) responded significantly stronger to surgery, with five out of 11 requiring additional doses of detomidine and ketamine. There were no significant differences between groups in respiratory rate, heart rate, blood pressure, SpO_2_ or blood gases. SpO_2_ levels <94 % were recorded in several pigs in both groups. No post-injection complications were reported at follow-up.

**Conclusions:**

The results from this study showed that lumbo-sacral epidural anaesthesia with lidocaine could successfully be administered during dissociative anaesthesia of livestock pigs by a veterinary student and without reported post-injection complications. It improved analgesia and anaesthesia during herniorrhaphy of sufficient duration to enable surgical skills training. The risks and consequences of hypoxaemia and hypoventilation should be considered.

## Background

In the European Union (EU) only few substances are available for sedation and anaesthesia in livestock pigs. Regulations prescribe that all substances used need to be licensed for the species or authorised through the cascade principle [1, 2] and the substance has to be validated and listed in appendix I of the EU Commission regulation [3]. Azaperone, detomidine, butorphanol and ketamine currently meet those requirements. Unfortunately, some pigs anaesthetised using a combination of these substances never reach a surgical plane of anaesthesia [4]. In addition, the anaesthesia is characterised by unpredictable duration and abrupt awakenings (Marie Sterning, SLU, Uppsala, Sweden, personal communication 2014). In Sweden, scrotal or inguinal herniorrhaphy of live-stock pigs in the field has traditionally been an important part of the surgical skills training of veterinary students. Due to the variable depth and length of dissociative anaesthesia when using the above listed, approved substances the number of pigs anesthetised in the field for surgical procedures to be performed by students has decreased. Thus, to ensure animal welfare during *e.g*. scrotal herniorrhaphy, the protocol needs modification.

Lumbo-sacral epidural administration of analgesic or local anaesthetic agents is widely used in hospital-based surgery in several species to improve intra- and post-operative analgesia of the abdomen and the hind limbs [5–8]. Local anaesthetics, for example lidocaine, administered epidurally provide desensitisation of the caudal abdomen and the hind limbs for up to 60 min [9]. In livestock pigs, lidocaine may be used according to the cascade principle or, in some countries, with a temporary general permit for all veterinarians. Publications of non-hospital use of lumbo-sacral epidural lidocaine in farm animals are limited [10]. To our knowledge, a protocol combining dissociative and lumbo-sacral epidural anaesthesia has not previously been evaluated in a field situation with livestock pigs.

The aims of the present study were to evaluate whether lumbo-sacral anaesthesia can be safely and successfully used in the field by a veterinary student and whether the combination of dissociative and lumbo-sacral epidural anaesthesia improves analgesia and anaesthesia to guarantee animal welfare during herniorrhaphy in livestock pigs enabling surgical skills training.

## Methods

### Study design

The study was designed as a prospective, randomised, blind experiment fulfilling all legal requirements for animal research in Sweden and was performed at a commercial pig-producing farm in Sweden. Approval was obtained from the Ethical Committee for Animal Research in Uppsala, Sweden (C154/9). A fifth-year veterinary student made all epidural injections and performed the surgery.

The owner was informed about the project, gave written consent for the farm’s pigs to be used in the study and did not benefit financially from participating in the project.

### Pigs

All 6–10-weeks-old, American Society of Anesthesiologist physical status I-II, non-castrated pigs with or without a scrotal hernia observed in the stables on study days were included in the study. The pigs were mixed breed Swedish Landrace-Yorkshire-Hampshire or Swedish Landrace-Yorkshire-Duroc with a mean weight (SD) of 14.3 (±3.4) kg. They were randomly allocated (by picking coded notes from a jar) to either a group receiving an active treatment (epidural lidocaine, group L) or a control group receiving a non-active substance (epidural saline, group C). Field conditions were authentic in that daily routines such as feeding continued as usual and the pigs were not subjected to starvation before anaesthesia.

### Preparation for anaesthesia

The pigs were visually inspected in order to detect obvious disease, which was cause for exclusion. Body weight of each pig was estimated and the pig was sedated with 4 mg/kg azaperone (Stresnil vet®, 40 mg/mL, Janssen Pharmaceutical, Belgium) intramuscularly (IM) in the neck muscle caudal to the base of the ear. The pig was left alone for 15 min in the pig house aisle with the lights switched off. It was then placed in a bag and weighed using a spring balance. If the bodyweight exceeded the estimated weight, the pig was given an additional injection of azaperone in order to reach 4 mg/kg. Clinical examination including auscultation of heart and lungs, determination of heart rate (HR) and respiratory rate (RR), inspection of mucus membranes and determination of body temperature (BT) per rectum was performed in the sedated pig.

### Anaesthesia

General anaesthesia was induced in the sedated pig immediately after the clinical examination. Detomidine 0.1 mg/kg (Domosedan vet® 10 mg/ml, Orion Pharma Animal Health, Sweden), butorphanol 0.2 mg/kg (Dolorex vet® 10 mg/ml, Intervet, Sweden) and ketamine 10 mg/kg (Ketaminol vet® 100 mg/mL, Intervet, Sweden) were given separately IM in the neck muscle using a 0.6 × 25 mm cannula. The pig also received an IM injection of ketoprofen 3 mg/kg (Romefen vet® 100 mg/mL, Merial Norden, Denmark) and was again left alone in the aisle. After 10 min, the anaesthetised pig was transported to a small room within the pig house, where the epidural injection and surgery were performed. Pigs reacting to handling or surgery were given an additional injection of detomidine and ketamine preferentially intravenously (IV) but if this failed the IM route was used. If the reaction was exhibited before suturing the inguinal canal, 50 % of the original dose was administered. A reaction after suturing the inguinal canal resulted in a bolus injection at 33 % of the original dose, after which no surgical activity was performed for five minutes. If the pig still reacted to castration of the second testicle, analgesia was improved by infiltrating 20–40 mg 2 % lidocaine intratesticularly.

The temperature in the temporary theatre varied between 19.9 and 24.6 °C.

### Epidural injection

The pig was placed in sternal recumbency on a metal table covered with a folded cloth, with the hips flexed and the hind legs extended alongside the abdomen. The length of the vertebral column was measured from the external occipital process to the first vertebra of the tail in order to calculate the doses for epidural injection [11] 1 ml for the first 40 cm of vertebral column, and another 0.15 ml for each additional cm. The lumbo-sacral area was aseptically prepared, although the hair was not clipped. Wearing sterile gloves, a needle (BD Microlance™ 3, 0.9 mm × 40 mm, BectonDickinson and Company, USA) was placed in the extradural space at a 90° angle to the back between the last lumbar vertebrae and the first sacral vertebrae. Correct placement was confirmed when no blood or liquor was obtained on aspiration and loss of resistance occurred on test-injecting saline.

In group L, 2 % lidocaine (Xylocain®, AstraZeneca, Sweden) was administered and group C received physiological saline. The injected volume was delivered over one minute. The containers of both substances were masked identically and not identifiable to the anaesthesiologist or the surgeon.

### Surgery

One minute after the epidural injection, the pig was turned into dorsal recumbency and the inguinal area prepared for surgery. Five minutes after the epidural injection, an approximately 10 cm long skin incision was made in the groin, above the inguinal canal. After manually detaching the testicle from the scrotum, the intestines were repositioned back into the abdomen. The spermatic cord was torqued, clamped with a haemostat and ligated with a transfixed ligature (Vetafil® 0.4 mm, WDT, Germany). At this point the hind end of the table was raised from horizontal to 10° to prevent the intestines from falling back into the hernia. The inguinal canal was closed with a continuous suture (Vetafil® 0.4 mm) and then the short end of the table was replaced in its original position and the skin was sutured with 2–3 isolated stitches (Vetafil® 0.3 mm, WDT, Germany). Surgery followed a specific time schedule, to mimic a possible surgical skills training event by a fifth-year veterinary student (see below).

Immediately after suture of the skin closed castration of the contra-lateral side was performed. The spermatic cord was ligated using a transfixed ligature (Vetafil® 0.4 mm). The castration was performed without intermission. The wound was left for second intention healing*.*

If having a scrotal hernia this was repaired on the affected side and routine castration was performed on the unaffected side. If the pig was an intact male without a hernia, castration of one side was performed using the scrotal hernia surgery technique.

Time schedule for the procedure:

-10 min (T-10): Preparation for epidural injection, ventral recumbency.
-5 min (T-5). Epidural injection.
-3 min (T-3). After epidural injection, after being turned to dorsal recumbency.
- 0 min (T0). Skin incision.
- 7 min (T7). Detachment of testis from scrotal wall.
- 10 min (T10). Clamping of spermatic cord. Table hind end elevated.
- 12 min (T12). Ligation of spermatic cord. Table hind end elevated.
- 17 min (T17). Suture of inguinal canal. Table hind end lowered after suture.
- 30 min (T30). Suture of the skin.
- Castration of other testicle, uninterrupted procedure.

Bleeding control after castration served as endpoint for surgery.

After surgery, the pig was marked in the ear for post-operative identification by the farmer and was left to recover in a small straw-filled carriage. When able to walk, it was returned to the aisle of its pen, where it stayed until the day after surgery before being returned to its home pen.

### Collection of data

Heart rate, RR, nociceptive responses (pedal withdrawal reflex, PWR; nasal septum pinch, NSP), sensitivity of the snout, palpebral reflex, nystagmus and muscular relaxation (jaw and forelimbs) were monitored manually by an experienced anaesthesiologist from preparation for epidural injection (T-10) until completion of surgery. PWR and NSP were tested using a haemostat maximally clamped to the first hatchet. Sensitivity of the snout was tested by gently stroking the snout with the index finger.

Reaction to surgery (change in breathing pattern, vocalisation or motions) was continuously monitored. The reactions were graded as positive or negative.

During the procedure, non-invasive systolic, diastolic and median blood pressure (SAP, DAP, MAP) was measured oscillometrically using a cuff placed above the carpus of a forelimb (Cardell® Veterinary Monitor 9402, Sharn Veterinary Inc. Tampa, Florida, USA). A pulse oximetry probe was placed on one of the pig’s ears or tongue and connected to the same machine as for blood pressure (BP). Physiological variables are reported at 5-min intervals from T-10 to T35.

At T5 and T30, arterial blood was collected anaerobically from 8 pigs in group C and 7 pigs in group L. The samples were processed immediately using a portable analyser and cartridges (i-STAT®1 Portable Clinical Analyzer, cartridge CG4+, Abbott Laboratories, Illinois, USA). Analyses included measurement of partial pressure of arterial oxygen (PaO_2_) and carbon dioxide (PaCO_2_) and pH, and calculated values of haemoglobin oxygen saturation (SaO_2_) at 37 °C. Body temperature was measured rectally in the azaperone-sedated pig and at the end of surgery, using a digital thermometer. Time for recovery to sternal recumbency and standing was only roughly noted.

### Interpretation of data

On responding positively to a stimulus with a dichotomous outcome, the pig was registered as a responder and thereafter considered a responder for the remainder of the procedure. This type of registration is denoted ‘accumulated frequency’. Hypoxaemia was defined as a SpO_2_ < 90 %.

### Statistics

Statistical analyses were carried out on all measured variables. For variables with a count outcome (nociceptive responses, sensitivity of the snout, palpebral reflex, nystagmus, muscular relaxation and iteration), Fisher’s exact test (1-sided) was employed. Body temperature, body weight, PaO_2_, PaCO_2,_ pH, surgery time and time to recovery were analysed with t-tests (2-sided).

Variables with a continuous outcome (DAP, MAP, SAP and SpO_2_) were analysed with repeated measurement ANOVA. In addition, differences between the treatment group (L) and the control group (C) were tested at each timepoint by Šidák-adjusted t-test. Šidák adjustment was also employed for within-group comparisons between each timepoint and the value at T0 (start of surgery).

Values were considered significant at *P <* 0.05. Results are presented as mean ± standard deviation (SD). All statistical calculations were performed using Stata 12.1 (StataCorp. 2011. Stata Statistical Software: Release 12. College Station, TX: StataCorp LP, Texas, USA).

## Results

A total of 22 pigs were included in the study, with 11 pigs each in groups C and L. The average weight was 14.1 ± 4.7 kg in group C and 14.6 ± 1.5 kg in group L, with no difference between groups. The volumes administered epidurally were 0.28 ± 0.02 ml/kg in group C and 0.29 ± 0.02 ml/kg in group L, equivalent to a dose of 5.7 ± 0.5 mg/kg. Time from administration of ketamine until first incision was 28 ± 1 min and 27 ± 3 min in group C and L, respectively.

**Fig. 1:**
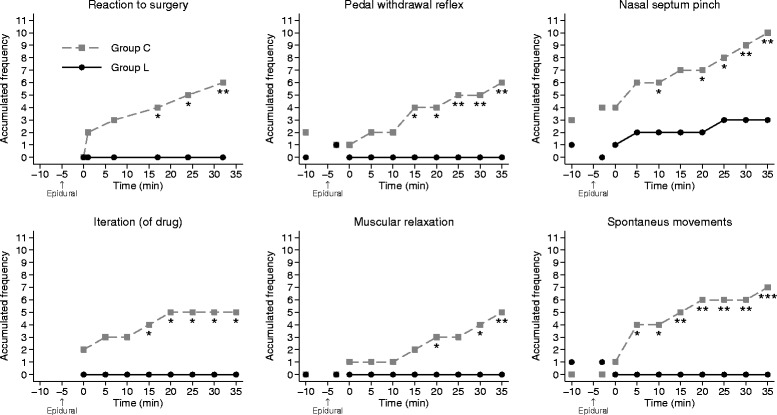
Differences between Group C and Group L in variables indicating anaesthetic depth and analgesia. Accumulated frequencies of the variables: Response to surgery, pedal withdrawal reflex, nasal septum pinch, iteration of drugs (detomidine-ketamine), muscular relaxation of forelimbs and spontaneous movement; during herniorrhaphy in pigs receiving an epidural injection of lidocaine (Group L: black rhombus) or an equivalent volume of physiological saline (Group C: grey squares) during azaperone-detomidine-butorphanol-ketamine anaesthesia. The X-axis shows the time in minutes relative to start of surgery at time 0. The time for epidural injection is indicated with an arrow below the X-axis. Significance levels between groups: * = *P <* 0.05, ** = *P <* 0.01, *** = *P <* 0.001

There were significant differences between the groups in regard to the accumulated frequency of the variables PWR, NSP, muscular relaxation of forelimbs, spontaneous movements and reaction to surgery with more responders in group C (Fig. 1). No pig in group L reacted to surgery during herniorrhaphy, whereas three group C pigs reacted to surgery on more than one occasion. One group C pig and one group L pig reacted at castration of the second testicle (not shown in Fig. 1). The pig in group L only showed a minor, transient change in breathing pattern.

The variables nystagmus, palpebral reflex and snout sensibility did not differ between the groups.

In group C five pigs received one additional injection of detomidine and ketamine and one pig received two additional injections, at 50 % of the initial dose, due to response to surgery. No pig in group L received an extra dose of anaesthetics. Three pigs in group C were administered lidocaine locally to enable completion of surgery, two of which had previously received additional ketamine-detomidine. No pig in group L received local administration of lidocaine. Significantly more pigs in group C received additional ketamine-detomidine and/or local lidocaine (*P <* 0.01).

Body temperature decreased similarly in both groups from pre-anaesthesia to post-surgery, from 38.8 ± 0.4 °C to 36.1 ± 1.0 °C in group C (*P <* 0.001) and from 39.1 ± 0.5 °C to 36.8 ± 1.0 °C in group L (*P <* 0.001).

**Fig. 2:**
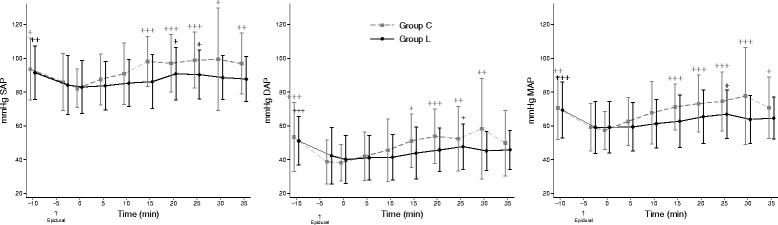
Systolic, diastolic and mean arterial systemic blood pressures. Mean values ± SD for systemic, diastolic and mean arterial blood pressure (SAP, DAP, MAP) during herniorrhaphy in pigs receiving an epidural injection of lidocaine (Group L: black rhombus) or an equivalent volume of physiological saline (Group C: grey squares) during azaperone-detomidine-butorphanol-ketamine anaesthesia. The X-axis shows the time in minutes relative to start of surgery at time 0. The time for epidural injection is indicated with an arrow below the X-axis. Recordings are shown until completion of herniorrhaphy at 35 min after incision. Significant differences over time compared with time 0 are denoted + (*P <* 0.05), ++ (*P <* 0.001), or +++ (*P <* 0.0001)

**Fig. 3:**
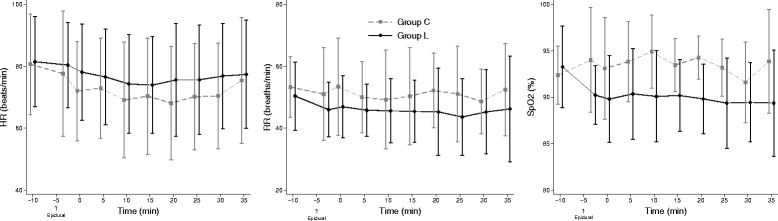
Heart rate, respiratory rate and SpO_2_. Mean values ± SD for heart rate (HR), respiratory rate (RR) and SpO_2_ during herniorrhaphy in pigs receiving an epidural injection of lidocaine (group L: black rhombus) or an equivalent volume of physiological saline (group C: grey squares) during azaperone-detomidine-butorphanol-ketamine anaesthesia. The X-axis shows the time in minutes relative to start of surgery at time 0. The time for epidural injection is indicated with an arrow below the X-axis. Recordings are shown until completion of herniorrhaphy at 35 min after incision. Significant differences over time compared with time 0 are denoted + (*P <* 0.05), ++ (*P <* 0.001), or +++ (*P <* 0.0001)

Heart rate, RR, SAP, MAP and DAP did not differ between the groups (Figs. 2 and 3). Mean arterial blood pressure was <60 mmHg during at least half of the procedure in two of the 11 pigs in group C and in four of the 11 pigs in group L.

There was a tendency for a general difference between groups as regards SpO_2_ (*P =* 0.06), but after Šidák correction the *post hoc* analysis could not identify any specific timepoint at which the mean values differed (Fig. 3). SpO_2_ values below 90 % at one or more sampling points were registered in 5/11 and 7/11 pigs in Group C and L respectively, and at 16 out of 113 sampling points in group C and at 44 out of 115 sampling points in Group L. Between T-10 and T0, 3/11 pigs in group C and 8/11 pigs in group L showed decreasing values for SpO_2_, but no significant changes over time were recorded in either group.

Mean values (±SD) for PaO_2_, PaCO_2_, SaO_2_ and pH are shown in Table 1. Paired arterial samples were only successfully obtained and analysed in five pigs in group C and in seven pigs in group L. Mean values were calculated on all samples, but statistics over time were only performed on paired samples. There were no significant differences in PaO_2_ or PaCO_2_ between groups. PaO_2_ decreased over time in group L (*P =* 0.02) but not in group C (*P =* 0.94). A PaO_2_ ≤ 8 kPa (and SaO_2_ ≤ 90 %) was measured in 3/13 samples from group C and in 4/14 samples from group L. A small but statistically significant increase was observed in PaCO_2_ in group C (*P =* 0.02). No difference over time was detected in group L (*P =* 0.25). There was a significant difference in arterial pH between groups at both sampling timepoints (*p* < 0.01 at T5 and *P =* 0.03 at T30), but there was no significant change over time within groups.

**Table 1:** Partial pressures of O_2_ and CO_2_, saturation of oxygen and pH in arterial blood

		Timepoint	
Variable	Group	T5	T30
PaO_2_ (kPa)	C	9.6 ± 2.2	10.1 ± 1.9
	L	10.0 ± 1.7	8.5 ± 1.9
PaCO_2_ (kPa)	C	6.2 ± 1.0	6.3 ± 0.6
	L	6.8 ± 0.5	7.2 ± 1.0
SaO_2_ (%)	C	92 ± 6	94 ± 3
		94 (82–97)	95 (88–97)
	L	92 ± 3	87 ± 10
		92 (88–96)	93 (68–95)
pH	C	7.38 ± 0.04	7.38 ± 0.03
	L	7.32 ± 0.01**	7.32 ± 0.05*

Mean values (±SD) at 5 and 30 min after start of surgery (T5 and T30) in control pigs (group C, *n* = 8) and in pigs receiving epidural lidocaine (group L, *n* = 7) during azaperone-detomidine-butorphanol-ketamine anaesthesia. PaO_2_: partial pressure of arterial oxygen; PaCO_2_: partial pressure of arterial carbon dioxide; SaO_2_: arterial oxygen saturation of haemoglobin. Significant differences between groups: * = *P <* 0.05; ** = *P <* 0.01. Significant differences to T5 are denoted (=*P <* 0.05)

Total surgery time (from incision until end of castration) was 41 ± 3 min in group C and 39 ± 5 min in group L. The *approximate* time from end of surgery until the pig was able to run was 76 ± 33 min in group C (*n* = 10) and 87 ± 33 min in group L (*n* = 9). No statistical comparison was performed on recovery times since the exact recovery times were not recorded.

All pigs recovered uneventfully from surgery and were returned to their home pens on the morning after surgery. No complication associated with surgery or the epidural injection was reported by the farmer at follow-up.

## Discussion

Comparing the frequency of intra-operative responses to noxious stimuli in the present study, dissociative anaesthesia combined with epidural administration of lidocaine was superior to dissociative anaesthesia alone during field surgery in pigs. It provided additional analgesia of sufficient degree and duration to guarantee animal welfare during scrotal herniorrhaphy and no post-injection complications were reported. It also proved a functional protocol for a teaching situation in which veterinary students practise their surgical skills. In the tested group no further clinically significant negative effects on the cardiovascular system were registered, although the results indicated a slightly more negative effect on gas exchange compared with dissociative anaesthesia alone. Thus the protocol appears to provide acceptable safety margins for field surgery in livestock pigs.

Seven out of 11 pigs in the control group reacted to surgery, during a surgical procedure shorter than 40 min. Among those pigs, two reacted at the initial incision of the skin and were obviously not at a level of anaesthesia profound enough even for superficial surgery. Using a similar protocol as for the control group in the present study, Heinonen *et al.* [4] concluded that dissociative anaesthesia was unreliable in pigs, but can be used for minor surgery. In that study, one-third of the pigs never reached a surgical level of anaesthesia and the duration of anaesthesia showed great individual variation. In contrast, a protocol composed of medetomidine-ketamine-butorphanol resulted in excellent surgical conditions [12]. However, medetomidine is not available for use in production animals in the European Union. Lumbo-sacral epidural anaesthesia is frequently used in in-hospital surgical procedures to provide analgesia of the caudal abdomen and was therefore added to the anaesthesia protocol approved for livestock pigs. The single pig in group L that reacted to surgery did so 45 min after epidural injection, when the effect of lidocaine had most likely begun to wear off. The reported duration of epidural anaesthesia using lidocaine in pigs varies between 30 and 60 min [9, 13]. The present protocol was developed to mimic a situation where final-year veterinary students perform herniorrhaphy as part of their surgical training. Therefore, the timing of events was slower than if surgery had been performed by an experienced veterinarian. Despite this, surgical conditions were excellent for most of the procedure in group L. In a routine situation, when a skilled veterinarian performs hernia surgery, it is usually completed within 20 min, so the duration of epidural lidocaine is sufficient in most circumstances.

In the present study, apart from reducing the nociceptive stimuli from the surgery site, epidural lidocaine also enhanced the depth of anaesthesia, as indicated by the decreased response to nociceptive provocations of the forelimbs (PWR) and nasal septum (NSP). Previous studies have shown that epidural analgesia/anaesthesia reduces the requirement for anaesthetic or hypnotic agents in several species such as pigs [14], dogs [15, 16], horses [7] and humans [17–19]. This effect might be explained by indirect central effects of spinal deafferentation [19, 20].

Despite the obvious differences between groups in the physical response to noxious stimuli, no differences in BP, HR or RR were detected between groups, and time-related changes in BP were similar in both groups. Increases in BP and HR are commonly used as indicators of intra-operative nociception [21, 22]. However, since the groups were small and blood pressure only measured oscillometrically, minor differences in BP and HR may not have been detected.

Several pigs in both groups of the present study were hypoxaemic (SpO_2_ or SaO_2_ < 90 %) at one or more sampling points during dissociative anaesthesia. The mean values for PaO_2_ in this study were lower than in the medetomidine-ketamine-butorphanol anaesthetised pigs [12], but comparable to the values presented by Heinonen *et al*. [4]. The pigs in the present study were positioned in dorsal recumbency, in contrast to the laterally recumbent pigs in the study by Sakaguchi *et al.* [12], which may have contributed to hypoxaemia [23, 24]. In addition, these pigs were livestock pigs, in which subclinical disease may have been present, possibly affecting oxygen uptake. Hypoxaemia is a well-recognised sequel to anaesthesia, and therefore oxygen supplementation is often standard routine during in-hospital procedures in anaesthetised or deeply sedated animals. In the field, oxygen may be provided through an oxygen cylinder or an oxygen generator and delivered through a face mask. However, for economic reasons this is not commonly practised in Sweden. To mimic the field situation, oxygen was not administered to the pigs in the present study.

Epidural anaesthesia using lidocaine has been reported to induce depression of respiratory function, with increases in PaCO_2_ and decreases in PaO_2_ [9, 25]. However, in the present study, on the basis of the few arterial samples taken, hypoventilation was similar in both groups. It is possible that with the inclusion of more animals and analysis of more arterial blood gas samples, significant group differences would have been detected. A similar degree of hypoventilation during dissociative anaesthesia of pigs has been reported previously [4, 12]. Arterial blood pH was lower in group L but the lowest individual pH values were not consistently associated with the highest PaCO2 values, which may indicate a metabolic component of the acidaemia. Since plasma lactate concentration rise quickly in pigs during stress [26], such as caused by handling, this may have contributed to the low pH.

The pigs recovered well from their lidocaine-induced paresis and no pig showed prolonged loss of motor function beyond recovery from dissociative anaesthesia. Epidural injections are usually performed under sterile conditions to reduce the risk of introducing bacteria into the spinal canal. Despite the non-sterile conditions in the present study, no post-injection infections were reported for any of the 22 pigs. Detailed knowledge of anatomy and careful attention to correct placement of the needle also reduce the risk of complications. The results from this and another field study [10] indicate that the lumbosacral injection technique can be successfully used in a field situation.

## Conclusion

Based on the results of the present study, epidural administration of lidocaine can be recommended in pigs anaesthetised with azaperone-detomidine-butorphanol-ketamine, in order to improve the quality of anaesthesia and analgesia during field surgery and during surgical skills training by veterinary students. However, the pigs are at risk of developing hypoxaemia and provision of supplemental oxygen should be considered.
